# Is helmet therapy effective in positional plagiocephaly? A systematic review and proposal for an updated management algorithm

**DOI:** 10.1007/s00381-026-07306-9

**Published:** 2026-06-01

**Authors:** Daniela Castaño-Bustos, Mariana Agudelo-Arrieta, Maria Isabel Ocampo-Navia, Sergio Esteban Chacón-Valencia, Wilfran Pérez-Méndez, Manuel Vergara-Lago

**Affiliations:** 1 Neurosurgery Department and Research Group, Pontificia Universidad Javeriana, Bogotá, Colombia; 2 Department of Neurosurgery, Hospital Universitario San Ignacio, Bogotá, Colombia

**Keywords:** Cranial orthosis, Deformational plagiocephaly, Helmet therapy, Physical therapy, Positional

## Abstract

Positional plagiocephaly, also known as deformational or non-synostotic plagiocephaly, is a common cranial deformity in infancy resulting from sustained external mechanical forces acting on the developing skull. Its prevalence has increased in recent decades, paralleling widespread adoption of supine sleep positioning recommendations. Although generally considered a benign condition, uncertainty persists regarding optimal management strategies, particularly the role and timing of cranial orthotic therapy. Conservative approaches, including repositioning techniques and physical therapy, are frequently employed, while helmet therapy is typically considered for moderate to severe cases or when initial treatments fail. This systematic review summarizes current evidence on therapeutic interventions for positional plagiocephaly, focusing on cranial shape outcomes, modifiers of treatment response, and safety profiles. The available evidence suggests that helmet therapy appears to be associated with faster early correction of cranial asymmetry, particularly in infants with moderate-to-severe deformity. However, current data do not demonstrate clear long-term superiority over conservative management, as differences between treatment modalities tend to diminish over time. These findings support the interpretation that cranial orthotic therapy may primarily accelerate the rate of correction rather than fundamentally alter long-term cranial morphology. An updated, age- and severity-based diagnostic and management algorithm is proposed to support individualized, evidence-informed clinical decision-making. These findings should be interpreted in the context of the limited number of included studies and the observational nature of the available evidence.

## Introduction

Positional plagiocephaly (PP), also known as deformational or non-synostotic plagiocephaly, is characterized by asymmetric cranial vault deformation secondary to sustained external mechanical forces acting on the malleable infant skull [1]. Over recent decades, its prevalence has increased substantially, a trend that parallels the widespread implementation of supine sleep positioning recommendations aimed at reducing sudden infant death syndrome [2, 3]. Although these public health measures have been unequivocally successful, they have unintentionally altered early cranial loading patterns, favoring the development of posterior cranial flattening in susceptible infants. While the deformity is considered primarily aesthetic and typically non-life-threatening, it has generated concern among healthcare professionals and caregivers regarding its potential impact on neurodevelopment and overall well-being [4].

The clinical management of positional plagiocephaly remains a subject of debate, with current treatment strategies ranging from conservative approaches, such as repositioning techniques and physical therapy, to more structured interventions like cranial orthotic therapy [5, 6]. Although helmet therapy is often reserved for moderate to severe cases or those unresponsive to repositioning and physiotherapy, there is no universal consensus regarding its effectiveness, optimal initiation age, or long-term outcomes. Furthermore, variability in clinical practice and the absence of standardized guidelines contribute to uncertainty in therapeutic decision-making.

Evidence-based guidelines are essential to inform clinicians and ensure appropriate, cost-effective care tailored to the individual needs of each patient. Identifying the most effective therapeutic interventions not only has the potential to enhance treatment outcomes but may also help reduce unnecessary interventions and healthcare costs. In addition, clear recommendations could alleviate parental anxiety and support healthcare providers in offering consistent, evidence-informed advice.

Given the increasing prevalence of positional plagiocephaly and the clinical uncertainty surrounding its management, this systematic review aims to evaluate the available evidence on therapeutic approaches for this condition. By synthesizing the current literature, the review seeks to establish a foundation for informed clinical decisions and contribute to the development of a diagnostic-therapeutic algorithm that improves both cosmetic outcomes and overall patient care.

## Materials and methods

This systematic review was conducted to identify the most current and relevant evidence regarding the clinical management of non-synostotic positional plagiocephaly to support informed decision-making in pediatric care. This review adhered to the Preferred Reporting Items for Systematic Reviews and Meta-Analyses (PRISMA) guidelines [7]. A comprehensive search was performed in four electronic databases: PubMed/MEDLINE, Scopus, Embase, and Web of Science. The search strategy was based on the terms “*plagiocephaly*,” “*nonsynostotic*” OR “*cranial deformities*,” “*nonsynostotic*” OR “*positional plagiocephaly*” OR “*deformational plagiocephaly*” OR “*cranial asymmetry*” OR “*flat head syndrome*” AND “*orthotic devices*” OR “*helmet therapy*” OR “*cranial orthosis*” AND “*physical therapy*” OR “*repositioning therapy*” OR “*repositioning techniques*” OR “*postural changes*” AND “*cranial vault asymmetry index*” OR “*deformity correction*” OR “*cranial shape improvement*” OR “*neurodevelopment outcomes*.” This strategy combined controlled vocabulary (MeSH/Emtree terms, when available) and free-text terms related to positional plagiocephaly, helmet therapy, and conservative management. The complete search strategies for each database are provided in Supplementary Table 1.

We limited the results to peer-reviewed articles published from January 2015 through January 2025, in English or Spanish. Eligible studies encompassed randomized controlled trials, cohort studies, case-control studies, and primary comparative studies, involving infants aged 0 to 12 months diagnosed with positional plagiocephaly without significant comorbidities (neurological, musculoskeletal, or genetic). These studies compared conservative management approaches, such as observation, physical therapy, or repositioning therapy, with cranial orthotic treatment therapy. Studies had to report outcomes related to cranial shape correction, morbidity, side effects, or long-term development, with a minimum follow-up of 6 months. We excluded studies involving craniosynostosis or surgical interventions, as well as non-primary publications (including systematic reviews, meta-analyses, editorials, commentaries, and non-peer-reviewed documents). To avoid duplication of data and ensure methodological consistency, only primary studies were included in the analysis. Additional exclusion criteria included studies lacking quantitative data or relevant clinical outcomes, studies not focused on primary positional plagiocephaly, those with insufficient follow-up (<6 months), non-human studies, and articles not published in English or Spanish. Although systematic reviews and meta-analyses were excluded from the primary analysis, they were considered in the discussion to provide contextual support and facilitate interpretation of findings.

The screening process was conducted using the web-based platform Rayyan (QCRI) [8], which optimized the review workflow by facilitating the identification of relevant studies and the removal of duplicate records. Two independent reviewers screened the titles and abstracts of all identified articles to assess their eligibility based on predefined inclusion and exclusion criteria, with blinded assessment, and conflicts were resolved by a third reviewer. Full-text articles were then analyzed in detail.

Data extraction was carried out independently by two reviewers using a standardized form, collecting relevant information on study design, population characteristics, type of intervention, outcome measures (such as cranial vault asymmetry index or cephalic index), and follow-up data. A qualitative and quantitative descriptive synthesis of the results was conducted, without meta-analysis, as it was not feasible due to clinical and methodological heterogeneity among included studies. The methodological quality of the included studies was evaluated using the Joanna Briggs Institute (JBI) critical appraisal tools appropriate for each study design, focusing on clinical description, diagnostic methodology, intervention clarity, and follow-up, with appraisal results used to inform the interpretation of findings but not as exclusion criteria [9]. The results of the risk-of-bias assessment were used to inform the interpretation of findings and are presented in Supplementary Table 2.

## Results

### Study selection

**Fig. 1:**
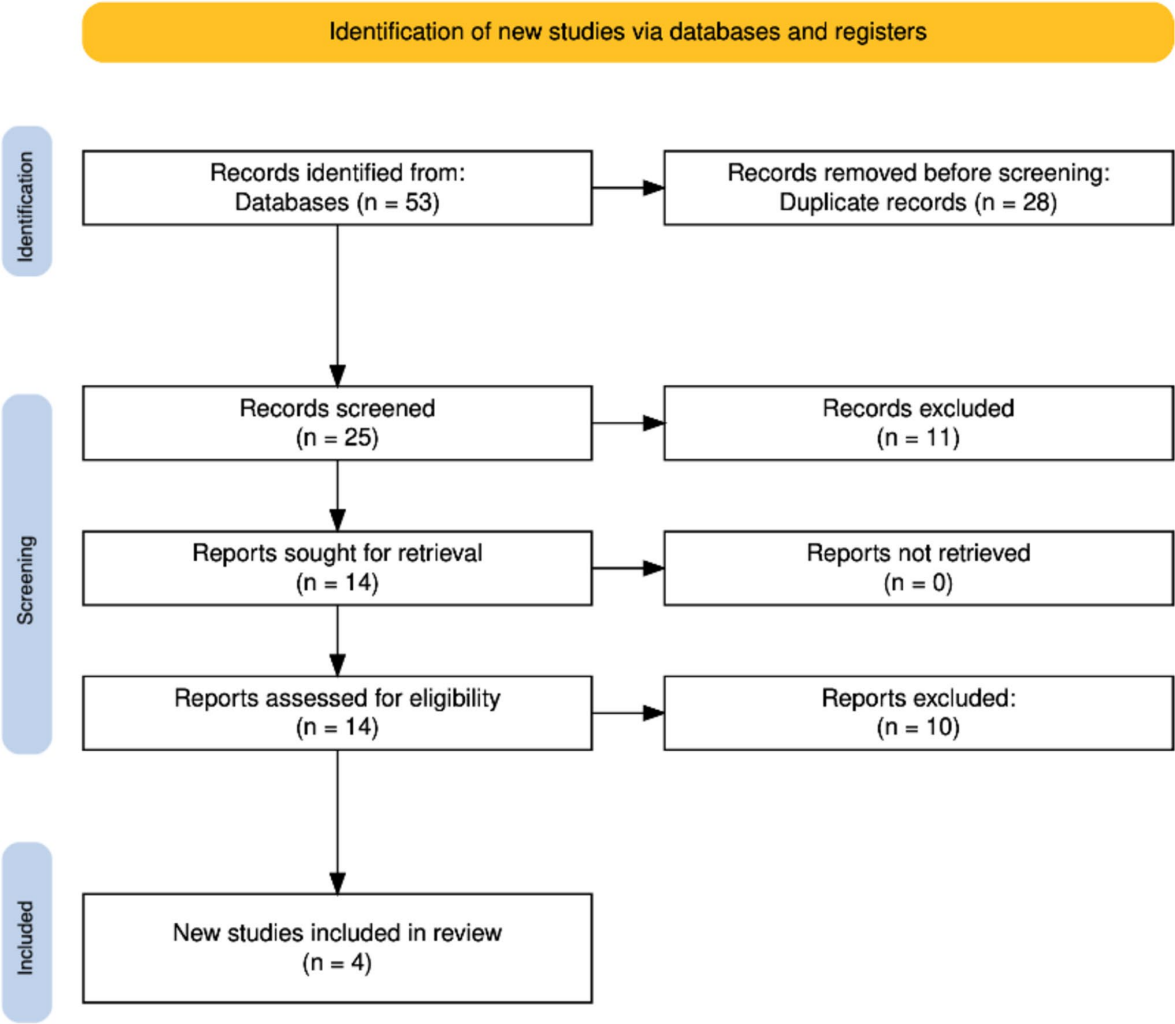
Inclusion flow diagram based on PRISMA 2020

The literature search identified studies evaluating therapeutic interventions for non-synostotic positional plagiocephaly. After removing duplicates and applying predefined eligibility criteria, relevant studies were selected for qualitative synthesis. The final sample included prospective and retrospective observational cohort studies published between January 2015 and January 2025. No randomized controlled trials met the inclusion criteria. The study selection process and reasons for exclusion at each stage are summarized in the PRISMA flow diagram (Fig. 1).

A total of 53 records were identified through database searching. After removal of 28 duplicates, 25 records underwent title and abstract screening. Of these, 11 were excluded, and 14 full-text articles were assessed for eligibility. Ten studies were excluded after full-text review for predefined reasons, and four studies were included in the qualitative synthesis. Detailed exclusion reasons are provided in Supplementary Table 3, including lack of comparator group, population outside the predefined age range, absence of relevant clinical outcomes, insufficient follow-up duration (<6 months), non-primary study design, and insufficient data for extraction. The small number of included studies and the absence of randomized controlled trials significantly limit the strength of the available evidence and should be considered when interpreting these findings.

### Study characteristics

The included studies originated from various geographic regions, thereby reflecting a range of clinical settings and practice patterns. All selected studies utilized observational cohort designs, encompassing both prospective and retrospective cohorts. The sample sizes exhibited considerable variation across the studies. A summary of the primary characteristics of the included studies is provided in Table 1.

**Table 1:** Characteristics of the included studies

Author (year)	Country	Study design	Sample size (*n*)	Overall mean age at baseline (months)	Follow-up (months)
Graham et al. (2024) [10]	USA	Prospective cohort study	34	3	12
Van Cruchten et al. (2022) [11]	Netherlands	Retrospective cohort study	58	3–14 (range)	60
Lam et al. (2017) [12]	USA	Retrospective cohort study	991	6.2	NR
Naidoo et al. (2015) [13]	USA	Prospective cohort study	100	4.6	53

All included studies assessed infants within their first year, which is the most critical period for cranial growth and shaping. Treatment typically started between 2 and 7 months old. Interventions involved repositioning techniques, structured physical therapy, cranial orthotic therapy, or combinations of these methods. Follow-up periods ranged from at least 6 months to long-term assessments lasting over 18 months in some studies.

Cranial shape outcomes were primarily evaluated using objective anthropometric measures, most commonly the cranial vault asymmetry index (CVAI) and the cephalic index (CI), which allowed for longitudinal assessment of treatment response.

### Cranial shape outcomes (therapy vs orthosis)

Across the included studies, cranial orthotic therapy tended to be associated with more rapid early improvement, with modest differences in magnitude in cranial shape compared with conservative management alone, particularly among infants with moderate-to-severe deformity at baseline. Reductions in the CVAI were more pronounced in helmet-treated cohorts, with reported mean decreases generally ranging from approximately 3.5 to 6.6%, whereas repositioning and physical therapy alone resulted in more modest reductions over comparable follow-up periods. These improvements appeared more evident when helmet therapy was initiated earlier and maintained for an adequate duration. The main cranial shape outcomes according to the therapeutic approach are summarized in Table 2.

**Table 2:** Therapeutic interventions and cranial outcomes in the included studies

Study	Intervention	Outcome	Age at initiation (months)	Baseline	Final	Mean change	Treatment duration (months)
Graham et al. (2024)	Repositioning	CVAI	2.38	7.26 ± 1.81	3.67 ± 1.86	−3.59	5.04
		CI	2.38	88.09 ± 3.62	86.51 ± 2.81	−1.58	5.04
	Helmet	CVAI	5.39	8.10 ± 3.63	3.66 ± 2.59	−4.50	4.7
		CI	5.39	91.26 ± 4.84	88.39 ± 3.20	−2.9	4.7
Van Cruchten et al. (2022)	Physical therapy	CI	5.9	91.80	85.9	−6.0	NR
	Helmet	CI	5.3	91.5	85.7	−5.8	NR
Lam et al. (2017)	Repositioning	CI	6.1	95.7 ± 3.0	93.8 ± 3.0	−2.0	NR
	Helmet	CI	6.4	97.7 ± 3.5	93.2 ± 3.0	−4.5	NR
Naidoo et al. (2015)	Repositioning	CVAI	4.42	8.3	5.05	−3.32	NR
		CI	4.42	90.3	85.4	−4.9	NR
	Helmet	CVAI	4.87	10.81	4.16	−6.65	3.1
		CI	4.87	92.7	84.9	−7.8	3.1

*CI*, cephalic index; *CVAI*, cranial vault asymmetry index; *NR*, not reported. A decrease in cephalic index (CI) reflects normalization toward age-adjusted values in brachycephalic patients. Values are reported as mean ± standard deviation when available; otherwise, values are presented as reported in the original studies

*Skin irritation and rash were reported in the helmet therapy group

Graham et al. reported a mean CVAI reduction of 4.5% with helmet therapy in comparison to 3.6% following repositioning therapy; meanwhile, Lam et al. observed more significant differences, with mean reductions of 6.6% and 3.3%, respectively [10, 12]. Collectively, these findings suggest a potentially greater degree of asymmetry correction achieved through orthotic treatment, especially among infants exhibiting more pronounced initial deformities.

Changes in CI followed a similar pattern. Helmet therapy was associated with average CI improvements ranging from approximately 2.9 to 7.8%, whereas conservative approaches yielded smaller or slower improvements, generally between 2.0 and 6.0% [11, 13]. Infants undergoing orthotic treatment exhibited earlier normalization of age-adjusted CI values, particularly when therapy was initiated before 6 months of age and sustained for an appropriate duration, most often between 3 and 6 months.

Baseline severity emerged as an important modifier of treatment response. Infants with greater initial cranial asymmetry experienced larger absolute reductions in CVAI with helmet therapy, although complete normalization was less consistently achieved in this subgroup. In contrast, infants with mild deformity frequently reached acceptable thresholds for cranial symmetry with conservative management alone, although over a longer time course [4].

In studies with extended follow-up beyond 12 to 24 months, differences in final CVAI and CI values between conservative and orthotic treatment groups tended to diminish, particularly among infants with mild-to-moderate baseline deformity. These findings suggest that cranial orthotic therapy may primarily accelerate early cranial shape correction rather than clearly modifying long-term cranial morphology, particularly in mild-to-moderate cases.

### Factors influencing treatment response

Multiple factors were consistently recognized as modifiers of treatment efficacy, as evidenced by their measurable influence on cranial morphology outcomes and the course of treatment. Age at the commencement of therapy emerged as a principal determinant of cranial remodeling capacity across all intervention types. Infants who initiated treatment prior to 5 to 6 months of age exhibited greater absolute reductions in CVAI, more rapid improvement trajectories, and higher rates of reaching established symmetry thresholds compared with those who commenced treatment later, irrespective of the treatment modality [10, 13].

The baseline severity of deformity influenced both the magnitude of cranial correction and the likelihood of complete normalization, as demonstrated by changes in CVAI and, when reported, also CI. Infants presenting with more severe initial asymmetry typically exhibited larger absolute reductions in CVAI during the course of treatment; however, normalization to age-adjusted reference values was less consistently achieved in this subgroup [6]. Conversely, infants with mild deformity demonstrated smaller absolute changes but a greater probability of attaining clinically acceptable symmetry criteria within shorter follow-up periods [6].

The presence of congenital muscular torticollis was correlated with less favorable cranial shape outcomes, including slower rates of CVAI reduction and extended duration to achievement stabilization [14]. Studies that reported treatment duration and response curves consistently showed improved cranial outcomes when cervical imbalance was addressed simultaneously, primarily through structured physical therapy. Additionally, treatment adherence factors, such as daily helmet wear time and total orthotic use, were directly linked to the extent of cranial shape improvement, highlighting adherence as an important factor influencing outcomes in helmet therapy groups [12, 13].

### Neurodevelopmental outcomes

Neurodevelopmental outcomes were assessed in a subset of included studies using standardized cognitive, motor, and language evaluation scales, usually administered during infancy or early toddlerhood [6]. Across these studies, no clear evidence supports a causal link between positional plagiocephaly and negative neurodevelopmental outcomes [10–13]. Measured developmental scores generally stayed within normal reference ranges, and when delays were reported, they were typically mild, temporary, and non-progressive.

Importantly, these developmental findings did not consistently correlate with the degree of cranial asymmetry or the extent of cranial shape correction achieved. Comparative analyses showed no consistent differences in neurodevelopmental outcomes between infants managed conservatively and those treated with cranial orthotic therapy. These observations suggest that positional plagiocephaly may serve as a clinical marker of developmental vulnerability, reflecting associated factors such as prematurity, torticollis, or limited prone positioning, rather than acting as an independent determinant of neurodevelopmental trajectory [12].

### Adverse effects and morbidity

Safety outcomes were mainly reported concerning cranial orthotic therapy, focusing on treatment-related morbidity and tolerability. In most studies, adverse effects were generally mild, localized, and self-limiting. The most frequently reported events included temporary skin irritation, pressure-related erythema, and localized discomfort, which were usually resolved by helmet adjustments, skin care measures, or brief pauses in use [12]. No serious adverse events, long-term complications, or treatment discontinuations due to morbidity were reported.

Conservative management strategies, including repositioning and physical therapy, were not associated with clinically relevant adverse effects. Overall, safety outcomes across treatment modalities indicate a favorable risk profile, supporting the use of both conservative and orthotic interventions when appropriately indicated and monitored. These findings should be interpreted in the context of the limited number of included studies and the observational nature of the available evidence. The included studies presented a moderate risk of bias, primarily related to confounding and the observational nature of the study designs. Selection and measurement biases were generally low to moderate, while confounding remained a consistent limitation across studies. These methodological considerations should be taken into account when interpreting the reported treatment effects.

## Discussion

### Diagnosis of positional plagiocephaly

Cranial deformities encompass a large group of different asymmetric skull shapes, which includes positional plagiocephaly, also known as “deformational plagiocephaly” or “posterior plagiocephaly” [6]. There is a wide variety of definitions, generating a broad conception of this entity. PP is a morphological change in the cranial vault, generated by external mechanical forces, leading to posterior asymmetrical flattening of the skull, without the presence of craniosynostosis [2, 5]. Diagnosis must be clinical by the detection of asymmetric head shape through physical examination, which should be considered the primary diagnostic approach, but may also include a head X-ray or a three-dimensional computed tomography (3D-CT) image in cases of questionable diagnosis to evaluate sutures and exclude other etiologies [15]. However, imaging studies, particularly those involving ionizing radiation, should be reserved for atypical or uncertain cases in which craniosynostosis cannot be confidently excluded. Routine imaging is not recommended in typical cases.

Severity classification depends on multiple measurement systems described nowadays. The most cost-effective is by using a diagonal caliper during examination and obtaining bilateral diagonal lengths of the head and calculating their difference (cranial vault asymmetry), but it is liable to human error and mismatch, which is defined by a value of >9 mm between both measures [1]. Other systems include the CVAI, calculated as the difference between the cranial diagonal diameters divided by the shorter diagonal diameter, multiplied by 100. This yields a percentage value that allows categorization of deformity severity into mild, moderate, and severe forms [1].

### Repositioning and physical therapy

Repositioning and physical therapy have traditionally been the first-line approach; however, there is a lack of standardized, detailed guidelines on their implementation. Persing et al. stipulated a plan for the prevention and management of positional skull deformities in infants, specifying that parents must alternate the infant’s position every time they put them to sleep, use supervised tummy time, spend the less time possible in car seats, and position the crib in a way that makes the infant look at the other way [16].

An exercise program was also proposed to be performed during each diaper change, consisting of three repetitions of head rotation toward each shoulder and lateral head tilting, with each position maintained for approximately 10 s. Other strategies include the “stool” technique for infants older than 3 months, in which the caregiver faces the infant while a second person gently rotates the chair to encourage active head turning [15–17]. Physical therapy shows evidence of reducing the prevalence of children aged 7 weeks and being more effective than repositioning in severe cases (class II evidence). There is still limited literature available to determine the time of initiation, duration, and type of exercises applied [18].

These conservative strategies remain the cornerstone of initial management, particularly in infants with mild deformity and early presentation.

### Cranial orthotic therapy (helmet therapy)

Cranial orthotic therapy remains one of the most debated interventions in the management of deformational plagiocephaly, largely due to the heterogeneity of available evidence and the absence of universally accepted criteria for its indication. While conservative measures such as repositioning and physical therapy are widely accepted as first-line interventions, the role of helmet therapy continues to generate discussion, particularly in infants with moderate to severe deformity or in those who present beyond the optimal window for conservative management [19].

The studies formally included in this review suggest that helmet therapy appears to be associated with faster and greater early correction of cranial asymmetry, particularly in moderate-to-severe cases; however, these findings should be interpreted in the context of the broader literature, the limited number of included studies, and the observational nature of the available evidence.

To ensure clarity, it is important to emphasize that certain studies discussed below were not part of the formal systematic review due to predefined eligibility criteria and are therefore not included in the qualitative synthesis. These studies are presented solely as external contextual evidence and should not be interpreted as equivalent to the analyzed dataset.

Within this context, a randomized controlled trial evaluating helmet therapy in infants aged 5 to 6 months with moderate-to-severe positional skull deformation demonstrated no significant differences in long-term cranial shape outcomes between helmet-treated infants and those managed according to the natural course at 24 months of follow-up [4]. The study also reported treatment-related adverse effects and highlighted the economic burden associated with helmet therapy, discouraging its routine use in otherwise healthy infants. These findings provide important context but must be interpreted cautiously, as they fall outside the predefined inclusion framework of this review. Rather than contradicting the included studies, they support the interpretation that helmet therapy primarily accelerates early cranial shape correction without clearly modifying long-term outcomes.

Consistent with the findings of the studies included in this review, observational and comparative studies suggest that helmet therapy may offer advantages in selected clinical scenarios. Cohort studies using objective anthropometric and three-dimensional cranial measurements have reported greater and more rapid reductions in cranial asymmetry indices, including CVAI and CI, in helmet-treated infants compared with those managed conservatively [20–22]. These benefits are most pronounced in infants with moderate-to-severe deformity and when treatment is initiated early, typically before 6 months of age. Collectively, these studies indicate that helmet therapy is associated with a greater and more rapid correction of cranial asymmetry, whereas conservative management generally results in slower improvement over time.

Taken together, these findings suggest that helmet therapy may primarily accelerate the rate of early cranial shape correction rather than fundamentally alter long-term cranial morphology.

Recent studies have emphasized that treatment outcomes are strongly influenced by patient and treatment-related factors rather than by the intervention alone. Age at initiation, baseline severity of deformity, presence of associated conditions such as congenital muscular torticollis, daily wear time, and total duration of therapy have all been identified as key determinants of success [23, 24]. Additionally, considerable variability exists in diagnostic criteria, severity classification systems, and outcome measures across studies, which limits direct comparability and contributes to the apparent inconsistency of reported results.

Positional plagiocephaly should be understood as a dynamic and often self-limiting condition, with substantial spontaneous improvement over time. This natural course represents a critical framework for interpreting treatment effects, as a considerable proportion of cranial remodeling occurs independently of intervention. Therefore, the clinical benefit of helmet therapy is more appropriately interpreted as a reduction in the time to correction rather than a modification of the final cranial outcome.

### Neurodevelopmental outcomes

Current evidence does not demonstrate a consistent association between positional plagiocephaly and adverse neurodevelopmental outcomes; however, this interpretation should be made cautiously given the observational nature of the available studies and the presence of potential confounding factors. No causal relationship can be established.

### Toward a new algorithmic approach

**Fig. 2:**
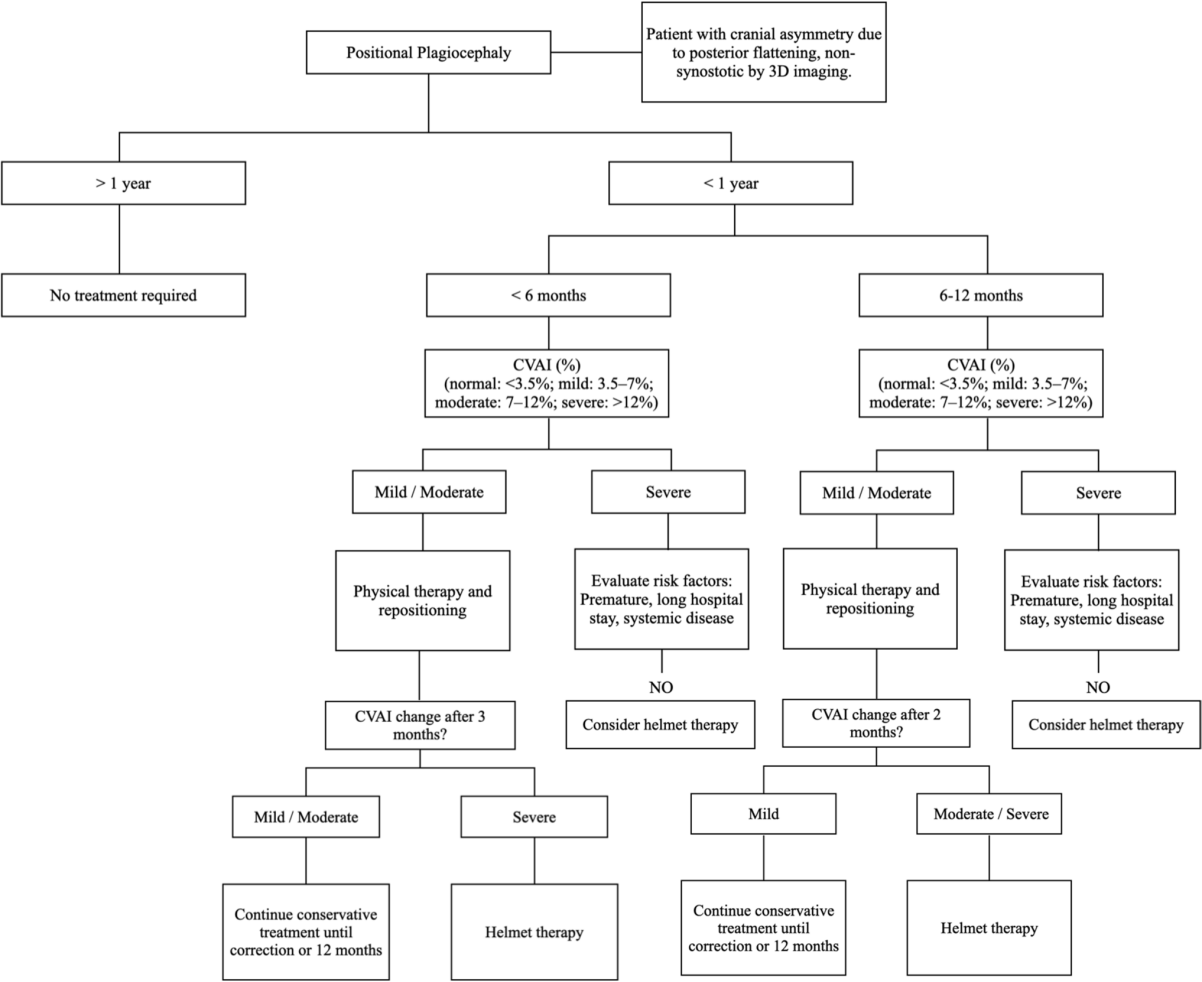
Algorithm for the clinical diagnosis and management of positional plagiocephaly based on age and severity. Source: authors’ own elaboration. CVAI: cranial vault asymmetry index

Based on the findings of the present review and aligned with current clinical practice, a proposed algorithmic approach for the management of deformational plagiocephaly is presented in Fig. 2.

### Limitations and future research directions

The evidence summarized in this review is predominantly derived from observational cohort studies, with limited randomized controlled data, which restricts definitive causal inference and increases the potential impact of confounding factors. Considerable heterogeneity was observed in study design, baseline severity classification, timing and duration of treatment, follow-up intervals, and outcome reporting. Variability in the use and reporting of cranial outcome measures such as the CVAI and CI limited direct comparisons across studies.

The limited number of studies meeting the predefined eligibility criteria also reflects the heterogeneity of the available literature and the scarcity of high-quality comparative studies with standardized outcome reporting. Additionally, the predominance of observational designs limits the ability to distinguish treatment effects from the natural course of cranial shape development.

Future research should focus on well-designed prospective studies using standardized definitions of deformity severity, uniform cranial measurements, and clearly reported treatment protocols. Comparative analyses stratified by baseline severity, along with long-term follow-up, are needed to better define the relative role of conservative versus orthotic management and to clarify whether helmet therapy modifies long-term outcomes or primarily accelerates early cranial shape correction.

## Conclusions

Positional plagiocephaly represents a frequent cranial deformation of infancy, whose increasing prevalence parallels current supine sleep positioning practices. Although it is generally considered a benign and non-life-threatening condition, its management remains heterogeneous due to variability in severity, timing of presentation, and the absence of universally standardized treatment guidelines.

The evidence reviewed suggests that conservative management, including repositioning strategies and physical therapy, is effective in many infants, particularly those with mild deformities and early presentation. These approaches are safe, non-invasive, and should be considered first-line treatment.

Cranial orthotic therapy is associated with faster early improvement in cranial asymmetry, especially in infants with moderate to severe deformity or in those who show limited response to conservative measures. However, current evidence indicates that long-term differences between treatment modalities tend to diminish over time, suggesting that helmet therapy may primarily accelerate correction rather than clearly modifying long-term outcomes.

Overall, treatment decisions should be individualized, taking into account the severity of deformity, age at presentation, response to initial conservative management, and family preferences. A stepwise, severity-based approach may help optimize outcomes while avoiding unnecessary interventions.

## Data Availability

No datasets were generated or analysed during the current study.

## Abbreviations

CI: Cephalic index
CVAI: Cranial vault asymmetry index
JBI: Joanna Briggs Institute
PP: Positional plagiocephaly
3D-CT: Three-dimensional computed tomography
